# Supplementary material for: A Modeled High-Density Fed-Batch Culture Improves Biomass Growth and β-Glucans Accumulation in Microchloropsis salina
Source: Plants (Basel). 2022 Nov 25;11(23):3229. doi: 10.3390/plants11233229 (PMC9738629; doi:10.3390/plants11233229)
Supplement: Supplementary file 1 [file plants-11-03229-s001.zip › Supplementary Figure S1.pdf]

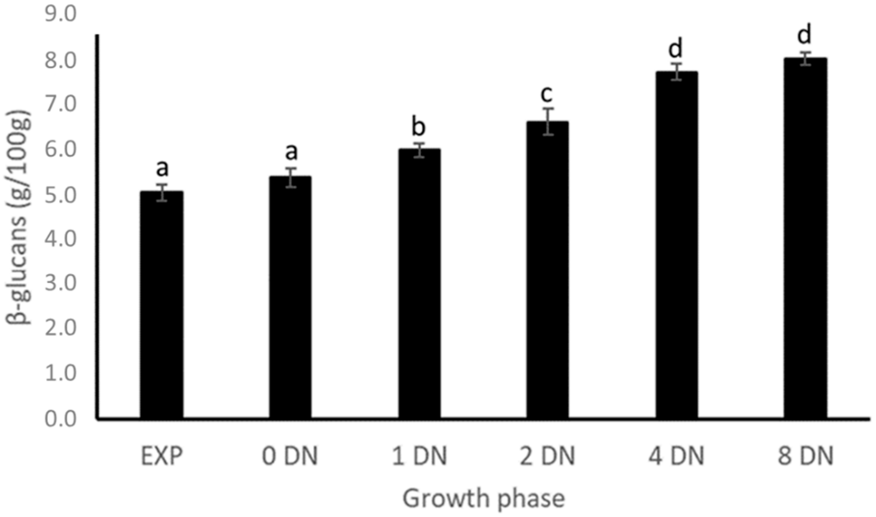

Supplementary Figure S1: Characterization of β-glucan production in high-density cultures of *M. salina* in different growth stages. The panels show the production of β-glucans in an exponential phase and at 0, 1, 2, 4, and 8 days after the total exhaustion of nitrogen in the culture medium, expressed in a percentage relation by the dry biomass of microalgae. ANOVA statistical analysis were performed ($p < 0.05$), "**a**","**b**","**c**", and "**d**" denotes significant differences between groups.
